# The complete mitochondrial genome of *Lambis chiragra*

**DOI:** 10.1080/23802359.2020.1775142

**Published:** 2020-06-08

**Authors:** Guoqiang Liu, Qibin Lao, Chunhua Zhang

**Affiliations:** Marine Environmental Monitoring Center of Beihai, State Oceanic Administration, Beihai, China

**Keywords:** *Lambis chiragra*, mitochondrial genome, phylogenetic relationship

## Abstract

In this study, we present the first complete mitochondrial genome sequence of the giant clam *Lambis chiragra*. The total length of the mitogenome is 16,404 bp. It contains the typical mitochondrial genomic structure, including 13 protein-coding genes, 22 transfer RNA genes, 2 ribosomal RNA genes, and 1 control region (D-loop). Mitogenome base composition is biased toward A + T content, at 66.4%. A phylogenetic tree based on complete mitogenome sequences revealed that, *L.chiragra* is the closest extant relative of *Conomurex luhuanus*.

Among the mollusks, the *Lambis* genus is one of the diverse group of gastropods (Mesogastropoda) in terms of species number as well as their wide range of distribution. The shells of *Lambis* gastropods show a wide variety of forms ranging from small-sized shells to large and elaborately ornamented with strongly flatted outer lip with radiating spines (Latiolais et al. 2006). *Lambis* species are long being commercially exploited from the sea for food, medicine, tools and ornaments. So far, these resources are largely looked as a source of commercial products which has resulted in overexploitation rather than appreciating their ecological values (Annamary and Mohanraj 2019). In this study, we sequenced the complete mitochondrial genome of *Lambis chiragra* to further the study of the taxonomy and phylogenetic relationships of Strombidae by increasing the amount of available molecular data.

The specimen was collected from Xisha, Hainan province, China (N16.70992, E112.35192). It was stored in Tropical Marine Biodiversity Collections of South China Sea(TMBC), Chinese Academy of Sciences, Guangzhou, China(specimen accession number: TMBC030691). The total genomic DNA was extracted following the modified CTAB DNA extraction protocol (Attitalla 2011), followed by library preps and pair-end sequencing (2 × 150 bp) with HiSeq (Illumina, San Diego, CA). Approximately 6145 Mb of raw data and 5683 Mb of clean data were obtained, and *de novo* assembled by the SOAP *de novo* software (Zhao et al. 2011) with an average of approximately 263 × coverage.

The mitogenome of *L. chiragra* is 16,404 bp in length (GenBank accession number MN885884). It contains 13 protein-coding genes (PCGs), 22 transfer RNA genes (tRNAs), 2 ribosomal RNA (*12S rRNA* and *16S rRNA*) genes, and a non-coding control region (D-loop). The mitogenome base composition of *L. chiragra* is biased toward A + T content at 66.4% (29.2% A, 37.2% T, 16.5% C, 17.1% G). The 13 identified PCGs vary in length from 159 to 1728 bp. Most protein-coding genes initiate with ATG except for ND2, ND4 and Cytb, which began with ATA, ATC or ATT. Ten protein-coding genes terminated with TAA whereas the ND2, ND1 and ND4L gene terminated with TAG. The lengths of the 22 tRNA genes range from 65 to 73 bp, and all of the tRNA genes have typical secondary structure. This (+) strand encodes for the cluster NARNI (trnN, trnA, trnR, trnN, and trnI) and trnS, trnD, trnV, trnL, trnL, trnP, trnS, trnH, trnF. The (−) strand encodes for the cluster MYCWQGE (trnM, trnY, trnC, trnW, trnQ, trnG, and trnE) and trnT. The *12S rRNA* gene is located between trnE-TTC and trnV-TAC, and is 982 bp long, while the *16S rRNA* gene is located between trnV-TAC and trnL-TAG, with a length of 1381 bp. A 1007 bp control region (D-loop) was located between *COIII* and trnF-GAA, with an A + T content of 68.03%.

A neighbor-joining phylogenetic tree of *L. chiragra* and other closely related species was constructed with the complete mitochondrial genomes using MEGA6 (Tamura et al. 2013) (Figure 1). The result showed that, *L.chiragra* is the closest extant relative of *Conomurex luhuanus*.

**Figure 1.**
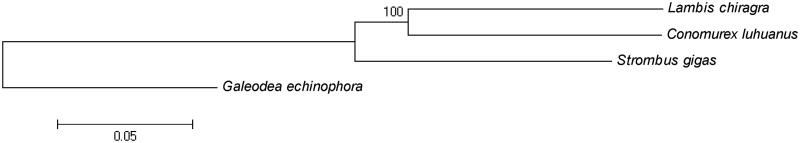
Neighbour-joining phylogenetic tree of *Lambis chiragra* and other 3 closely related species based on the complete mitochondrial genomes. GenBank accession numbers: *Conomurex luhuanus* (NC035726); *Strombus gigas* (KM245630); *Galeodea echinophora* (KP716635).

## Data Availability

The authors confirm that the data supporting the findings of this study are available within the article and its supplementary materials. https://www.ncbi.nlm.nih.gov/nuccore/MN885884
